# Intestinal helminthic infection and allergic disorders among school children enrolled in mass deworming program, Sululta, Ethiopia

**DOI:** 10.1186/s13223-021-00545-2

**Published:** 2021-04-23

**Authors:** Dessie Abera, Moges Wordofa, Abiyot Mesfin, Gemechu Tadesse, Mistire Wolde, Kassu Desta, Aster Tsegaye, Bineyam Taye

**Affiliations:** 1 Department of Medical Laboratory Sciences, Addis Ababa University, Addis Ababa, Ethiopia; 2 Korea Hospital, Addis Ababa, Ethiopia; 3 Ethiopian Public Health Institute, Addis Ababa, Ethiopia; 4 Department of Biology, Colgate University, 214 Olin Hall, 13 Oak Dr, Hamilton, NY USA

**Keywords:** Allergy, Intestinal helminths, School children

## Abstract

**Background:**

Intestinal helminths have been proposed to have a protective role against allergic sensitization and atopic diseases. However, consistent data demonstrating this are lacking in Sub-Saharan countries. We aimed to assess the association between intestinal helminths and allergic disorders among school children enrolled in mass deworming program in Sululta, Ethiopia.

**Methods:**

A cross sectional study was conducted among 526 school children aged 5 to 14 years old from primary government schools in Sululta district, Ethiopia. An interviewer-led questionnaire administered to parents provided information on demographic and lifestyle variables. Questions on allergic disease symptoms were collected using the International Study of Asthma and Allergies in Children (ISAAC) questionnaire 6 months following deworming treatments. Atopy was defined as a positive skin prick test reaction to one or both dust mite (*Dermatophagoides*) and German cockroach (*Blatella germanica*) allergens. Fresh stool samples were collected, processed, and examined by direct wet mount, Kato-Katz technique, and formol-ether concentration technique. Multivariate logistic regressions were used to assess the association between allergic disorder and helminths infection.

**Results:**

Of the total 526 school children, 58.2% were females. Overall, 24% (126/526) had allergic symptoms, 5.1% (27/526) had atopy, and 16.9% (89/526) had intestinal helminths. There was no association between helminthic infection and self-reported allergic symptoms (P = 0.317), but *Ascaris lumbricoides* infection was positively associated with atopy (AOR = 4.307, 95% CI 1.143–16.222, P = 0.031). Atopy was related to increased allergy symptoms (AOR = 2.787, 95% CI 1.253–6.197, P = 0.012), and family history of allergy was associated with increased childhood allergy (AOR = 2.753, 95% CI 1.565–4.841, P = 0.001). Deworming in the past 6 months showed a reduced odd of self-reported allergic symptoms (AOR = 0.581, 95% CI 0.366–0.954, P = 0.034).

**Conclusion:**

While no significant association between self-reported allergy and helminths was found in this study, this may have been due to the low prevalence and intensity of helminthic infection in the sample. There was a positive association between *Ascaris lumbricoides* and atopy. To further examine the underlying mechanism behind this positive association, a longitudinal study is needed.

## Background

The prevalence of allergic disorders, such as asthma, atopic dermatitis, and eczema, has significantly increased in recent decades and, as a result, these are now a major public health concern around the world. While the prevalence of allergic diseases tends to be lower in developing countries than in developed countries, this is starting to change, with the allergic diseases becoming more common in developing countries [1]. This rise has been highly linked to many factors, such as environmental allergens, changes in lifestyles, and a decline of bacterial infection, viral infection, and helminthic infection [2].

Currently, due to the increase in allergic diseases in developing countries, there is a strong curiosity among the scientific community about whether helminthic infection protects against or causes allergic diseases [3]. Numerous studies have shown that helminthic infection may be protective, playing a crucial role in shaping human health. This protective role is largely explained by the hygiene hypothesis. The original hygiene hypothesis promotes the idea that there is an inverse relationship between Th1 cells, which are associated with viruses and bacterial infections, and Th2 cells, which are associated with allergies. The hypothesis postulates that decreasing childhood infection leads to decreased Th1 cells, then allowing Th2 cells to proliferate and lead to increased allergic disease and sensitization [4]. However, the role of helminthic infection on hygiene hypothesis slightly different. Helminths exert their immunoregulatory actions by modulating cells of both the innate and adaptive immune systems. Regarding T-cells, helminths may promote a Th2-type response and downregulate Th1/Th17 differentiation, leading to increased Th2-type cytokine (IL-4, IL-5, IL-9, IL-10, IL-13) and decreased Th1/Th17-type cytokine (TNF-α, IFN-γ, IL-6, IL-12, IL-17) secretion [5]. A study among schoolchildren showed helminths infected lower levels of allergic reactivity than their uninfected classmates, although both the parasite and the allergen evoke Th2 responsiveness [6]. The authors suggested, infected children generated high levels of an immunosuppressive cytokine, interleukin-10 (IL-10), when their peripheral T cells were challenged with antigen from the Schistosoma parasite. However, others have also shown, the role of helminth infections on allergic disorders vary by type of helminths species, time of infestation, and the intensity of the infection [7, 8]. Furthermore, inconsistent epidemiological evidences have emerged, and helminths infections are associated with decreased prevalence of atopy [9–11], while others showed either positive, negative, or no relation between helminthic infection and allergic disease [12, 13].

While helminths infections may have a protective role in allergic disorders, they also have many proven detrimental effects on health and affect approximately 1.5 billion people worldwide. They are particularly high in low-income countries, where the allergic disease is low, and the reverse is true in developed countries [14, 15]. Due to these detrimental health effects, the World Health Organization (WHO) recommends controlling soil-transmitted helminths (STHs) infections in high-risk countries by implementing mass deworming programs. Mass deworming (i.e., irrespective of infection status) of school-aged children consist of a single-dose of an anthelmintic drug: either annually in areas with STH prevalence between 20 and 50%, or biannually in areas with STH prevalence above 50% [16]. Soil-transmitted helminths (STHs) comprise a number of intestinal nematodes such as *Ascaris lumbricoides*, Hookworm *(Ancylostoma duodenale and Necator americanus)* and *Trichuris trichiura.* However, mass deworming has been claimed to possibly increase the prevalence of the allergic disease, especially in areas endemic with helminthic infections [17]. There is little information about the association of helminthic infection and allergic disorders in the setting of mass deworming in Ethiopia. Hence, this study aimed to assess the association between helminthic infection and allergic disorders among school children enrolled in mass deworming program in Sululta, Ethiopia.

## Methods

### Study setting and design

This cross-sectional study was conducted from April 2017 to June 2017 among school children aged 5–14 years old in Sululta district, Ethiopia. Sululta is located in the North Showa zone, and one of the special zones of the Oromia regional state, surrounding Addis Ababa. It is 21 km from Addis Ababa, has a total area of 158km^2^ and it has a total population of 129,000. The district is semi-urban in which the majority of people are merchants, but subsistence farming is still practiced.

### Study subjects

A list of all government primary schools was obtained from the Sululta district education bureau; as a result, there are nine government primary schools and twenty-eight private primary schools. A random cluster sampling technique was used to select three primary schools out of the nine government primary schools and twenty- eight private primary schools. The three selected schools have a total of 3193 students**.** We requested the schools to participate with a letter of explanation to the principals and supervisors of the schools outlining the intention of the study and the procedures that were to be conducted. In addition, we obtained the contact information of the parents through the school and invited them to participate by sending them the same letter of explanation that was sent to the schools.

After we obtained written consent from parents/legal guardians, allocation of students was made proportional to the number of students in each school. To select the study participants, the students were first stratified according to their educational level (kg to grade 8) and then were taken from each class category by systematic random sampling using class roster as a sampling frame. Systematic random sampling technique was then used to select participants from each schools and grades. Information regarding deworming was obtained from the school principals. Accordingly, the deworming program was the first cycle in Sululta district and almost 81% of the students received anthelmintic treatment (500 mg of mebendazole) 6 months before this data collection.

### Sample size determination

The sample size (n) was estimated using the single population proportion formula taking 27.2% prevalence of intestinal helminths from a previous study in Babile, Ethiopia [18], with a marginal error of 4% at 95% confidence and a 1.96 standard score (z). Accordingly, the minimum sample size was determined to be 475 and to minimize errors arising from the likelihood of non-compliance the sample size was increased by 10%, which gave a final sample size of 523. Nevertheless, we enrolled 526 participants.

### Questionnaires

Written informed consent was obtained from the child and parents/legal guardians. An interviewer-administered questionnaire was used to interview parents/guardians about their socio-demographics, their daily habits, and household information. The questionnaire was modified from the international study of asthma and allergy childhood questionnaire (ISAAC) to assess allergic symptoms like skin itch, asthma, rhinitis, eczema, and wheeze in the past 12 months [19]. The questionnaire was translated to the national language (Amharic) and local language (Afaan Oromo), and it was pretested. Allergy assessment was done 6 months following deworming.

### Stool collection and examination

Instructions were given to children not to contaminate the stool with water and urine and then each child was provided a clean, dry, disinfectant-free, wide-mouth plastic container to collect about 20 g of stool specimen. Three parasitological tests were used to examine the presence of intestinal parasites: direct wet mount, Kato Katz technique, and ethyl-ether sedimentation. Direct wet mount of the sample was performed using a 0.85% saline solution at the study site within 30 min. A small portion of the stool samples was preserved with 3 ml of sodium acetate acetic acid formalin (SAF) per gram and transferred back to Addis Ababa University, College of Health Sciences, and Department of Medical Laboratory Sciences where the sedimentation technique was performed. In the sedimentation technique, one gram of fecal matter was dissolved in 7 ml of formalin and vortexed before being filtered through a gauze mesh. The resulting solution was transferred back to the centrifuge tube and combined with 4 ml of ethyl acetate, mixed thoroughly, then centrifuged at 2000 rpm for 2 min [20].

Kato-Katz was processed and examined at the school site on the same day of stool collection within 30 min. In the Kato Katz technique, 41.7 mg of sieved fecal material was placed on the slide by using a standardized template. Cellophane soaked overnight in methylene blue glycerol solution was placed over the sample before being pressed down and read. Moreover, eggs per gram (epg) of faeces were counted after 30 min and multiplied by 24 to determine the intensity of infection based on WHO grading system [15]. Kato-Katz was defined as a parasitological method to determine the presence of intestinal helminths and to quantify the number of eggs per gram of faeces.

### Atopic sensitization

Skin prick test (Imunotek Madrid, Spain) was performed using panel of two allergens: German cockroach (*Blattella germanica*) and dust mite (*Deatophagoidesrm*). Histamine and saline were used as positive and negative controls, respectively. After cleaning the forearm with 70% alcohol, a drop of each solution was placed on the volar surface of the forearm and the dermis was pierced using a metal lancet**.** Atopy was defined as positive for one or both allergens when the wheal diameter is greater than 3 mm than the negative control after 15 min of skin prick [21].

### Ethical clearance

Ethical approval was given by departmental Research and Ethics Review Committee (DRERC) of medical laboratory sciences, School of Allied Health Sciences, College of Health Sciences, Addis Ababa University. Children positive for intestinal helminths were treated with anthelminthic at the nearby health center.

### Statistical analysis

Data were entered using epidata software and then exported and analyzed using SPSS version 21. Descriptive statistics were performed to describe the socio-demographic characteristics and allergy symptoms of study participants. Our hypothesis that helminths infection would be associated with a lower prevalence of allergic disorders was assessed using multivariate logistic regression. We estimated odds ratios (ORs) associated with different types of helminths and allergic disorders using multivariate logistic regression. The ORs were adjusted for a priori and potential confounders such as socio-demographic characteristics. Probability values < 0.05 were considered statistically significant for main effects.

## Results

### Socio-demographic characteristics of study participants

A total of 526 school children participated from 3 primary schools and 58.2% of the students were females. The students’ ages ranged from 5 to 14 years old and their mean age was 10.8 years old. The majority of the students were in the age of 10–14 years 70% (368/526) and 57.4% (302/526) of students were in grades 1–4. Most of the students, 76% (400/526) live in semi-urban areas and 81.2% (427/526) of students received anthelmintic treatment in the last 6 months (before data collection). In regard to maternal and paternal educational level, 52.1% (274/526) of students’ mothers and 17.5% (92/526) of students’ fathers were illiterate. The majority of the students’ mothers were housewives, 58.6% (308/526) and 26.6% (138/526) of fathers were merchants (Table 1).

**Table 1:** Socio-demographic characteristics of children and their parents in selected primary schools in Sululta, Ethiopia, 2017 (n = 526)

Variables	Total number	Percent
Sex		
Male	220	41.8
Female	306	58.2
Age category in years		
5–9	158	30.0
10–14	368	70.0
Mother's education		
Illiterate	274	52.1
Write & read	92	17.5
Primary	110	20.9
High school	30	5.7
Higher education	20	3.8
Father's education		
Illiterate	92	17.5
Write &read	176	33.5
Primary	155	29.5
High school	74	14.1
Higher education	29	5.5
Residence		
Urban	400	76
Rural	126	24
Grade level		
Kg	59	11.2
1–4	302	57.4
5–8	165	31.4
Deworming status		
Yes	427	81.2
No	99	18.8
Maternal occupation		
Civil servant	35	6.7
House wife	308	58.6
Private	35	6.7
Farmer	12	2.3
Daily laborer	71	13.5
Merchant	65	12.4
Paternal occupation		
Civil servant	86	16.3
Private	98	18.6
Merchant	138	26.2
Daily laborer	129	24.5
Farmer	51	9.7
No work	24	4.6

### Prevalence of intestinal helminths, allergic symptoms, and atopy

Out of 526 school children, 16.9% were positive for at least one intestinal helminth. The most common intestinal helminths infection identified was *Hymenolepis nana,* found in 6.3% (33/526) of students, followed by *Ascaris lumbricoides,* found in 3.4% (18/526) (Table 2). Based on self-reported allergy symptoms in the study group, the overall prevalence of any allergy symptom was 24% (126/526). Skin itch and rhinitis were the most reported allergy symptoms, found in 18.8% (99/526), and 8.9% (47/526) respectively. The prevalence of atopy to cockroach or house dust mite allergen was 5.1% (27/526). Cockroach was the most common allergen with a prevalence of 2.7% (14/526), and reactivity to both house dust mite and cockroach was 1.7% (9/526), as depicted in (Table 2).

**Table 2:** Percentage distribution of intestinal helminths,allergy symptoms, and atopy among primary school childern in sululta, Ethiopia, 2017 (n = 526)

Variables	Number	Percent
Any helminths*	89	16.9
*Ascaris lumbricoids*	18	3.4
Hookworm	12	2.3
*Tricuris tricuria*	11	2.1
*Hymenolepis nana*	33	6.3
Any allergy**	126	24.0
Asthma		
Asthma ever	16	3.0
Wheeze in 12 months	46	8.7
Rhinitis	47	8.9
Eczema		
Eczema ever	28	5.3
Skin itch in 12 months	99	18.8
Atopy***	27	5.1
House dust mite alone	4	0.7
Cockroach alone	14	2.7
Both mite and Cockroach****	9	1.7

^*^Any helminths**:** Intestinal helminths, positive by direct wet mount, Kato-Katz or concentration

^**^Any allergy**:** allergy symptoms reported as, wheeze, eczema, asthma or skin itch

^***^Atopy**:** skin prick test positive either house dust mite or cockroach

Both****: if study subjects showed positive skin prick tests reaction for both allergens (House dust mite and cockroach)

### Association of allergic symptoms with intestinal helminthic infection

As indicated in (Table 3), among 89 intestinal helminthic infected children, 28.1% (25/89) had any allergic symptoms which was slightly higher than helminths negative children, in which 23.1% (101/437) had any allergic symptoms. There was no significant association between helminthic infection and self-reported allergic symptoms (P = 0.317). Individual parasites, *Ascaris lumbricoides, Trichuris trichuria,* and *Hymnelopis nana* had no significant association with self-reported allergic symptoms (P = 0.465, P = 0.269, P = 0.542), respectively. However, hookworm infection showed a positive significant association with self-reported allergic symptoms (AOR = 4.309, 95%CI = 1.321–14.056, P = 0.015).

**Table 3:** Association of any allergic symptoms with helminthic infection among primary school children in Sululta, Ethiopia, 2017 (n = 526)

Variables	Total	Any allergy^b^		COR	95% CI	P-value	AOR	95% CI	P-value
		Yes (%)	No (%)						
Any helminthes^a^									
Yes	89	25 (28.1)	64 (71.9)	1.30	0.778–2.171	0.317			
No	437	101 (23.1)	336 (76.9)	1					
*Ascaris lumbricoids*									
Yes	18	3 (16.7)	15 (83.3)	0.626	0.178–2.198	0.465			
No	508	123 (24.2)	385 (75.8)	1					
*Tricuris tricuria*									
Yes	11	1 (9.1)	10 (90.9)	0.312	0.040–2.461	0.269			
No	515	125 (24.3)	390 (75.7)	1					
*Hookworm*									
Yes	12	7 (58.3)	5(41.7)	4.677	1.448–14.910	0.010	4.39	1.321–14.056	0.015
No	514	119 (23.2)	395 (76.8)	1			1		
*Hymnelopis nana*									
Yes	33	4 (12.1)	29 (87.9)	0.705	0.194–2.203	0.542			
No	493	122 (24.7)	371 (75.3)	1					

COR: Crude odds ratio; AOR: adjusted odds ratio; CI: confidence interval

^a^Any helminths: Intestinal helminths, positive by direct wet mount, Kato-Katz or concentration

^b^Any allergy: allergy symptoms reported as, wheeze, eczema, asthma or skin itch

### Association of atopy with helminthic infection and allergic symptoms

Among helminths positive children, 6.7% (6/89) were positive for atopy, and among helminths negative children, 4.8% (21/437) were positive for atopy. Helminthic infection was not significantly related to atopy (P = 0.453). However, when subgroup analysis was performed between helminths species, there was a positive significant association between *Ascaris lumbricoids* infection and atopy (AOR = 4.307, 95%CI = 1.143–16.222, P = 0.031) as demonstrated in (Table 4).

**Table 4:** Association of atopy with intestinal helminthic infection and allergy symptoms among primary school children in Sululta, Ethiopia, 2017 (n = 526)

Variables		Atopy (yes %)	Atopy (no %)	COR	CI (95%)	P-value	AOR	CI (95%)	P-value
Any helminths									
Yes	89	6 (6.7)	83 (93.3)	1.432	0.561–3.656	0.453			
No	437	21 (4.8)	416 (95.2)	1					
*Ascaris lumbricoides*									
Yes	18	3 (16.7)	15 (83.3)	4.003	1.093–14.883	0.036	4.307	1.143–16.222	0.031
No	508	24 (4.7)	484 (95.3)	1					
Hookworm									
Yes	12	1 (8.3)	11 (91.7)	1.706	0.212–13.723	0.615			
No	514	26 (5.1)	488 (94.9)	1					
*Trichuris trichuria*									
Yes	11	1(9.1)	10 (90.9)	1.881	0.232–15.254	0.554			
No	515	26(5.0)	489 (95.0)	1					
*Hymnelopis nana*									
Yes	33	2 (6.1)	31 (93.9)	1.893	0.265–15.467	0.603			
No	493	25 (5.1)	468 (94.9)	1					
Any allergy									
Yes	126	12 (9.5)	114 (90.5)	2.702	1.229–5.937	0.013	2.843	1.281–6.307	0.010
No	400	15 (3.8)	385(96.2)	1					
Wheeze									
Yes	46	6 (13.0)	40 (87.0)	3.279	1.251–8.589	0.016	2.430	0.874–6.757	0.089
No	480	21 (4.4)	459 (95.6)	1					
Rhinitis									
Yes	47	4 (8.5)	43 (91.5)	1.844	0.610–5.579	0.278			
No	479	23 (4.8)	456 (95.2)	1					
Eczema									
Yes	28	2 (7.1)	26 (92.9)	1.455	0.327–6.480	0.622			
No	498	25 (5.0)	473 (95.0)	1					
Skin itch									
Yes	99	10 (10.1)	89 (89.9)	2.710	1.201–6.116	0.016	2.292	0.964–5.453	0.061
No	427	17 (4.0)	410 (96.0)	1					

Children who reported one or more allergic symptoms were more likely to be positive for atopy (AOR = 2.843, 95% CI 1.281–6.307, P = 0.010). Children who had a history of wheeze and skin itch showed a significant increased odds of atopy (COR = 3.279, 95% CI 1.251–8.589, P = 0.016, & COR = 2.710, 95% CI 1.201–6.116, P = 0.016) respectively, but the adjusted odds ratio in the multivariate analysis showed a non-significant relationship (Table 4).

### Potential risk factors for allergy

Male students reported slightly higher allergic symptoms, 25% (55/220) than female students 23% (71/306). However, the difference was not statistically significant (P = 0.634). Among children who had a history of animal contact, 24.6% (85/346) reported allergic symptoms but there was no significant association between allergic symptoms and animal contact (P = 0.648). Almost all children with allergic symptoms were from families who used charcoal as a source of fuel, 24% (125/521), but no statistically significant association was observed (P = 0.835). Out of 27 skin prick test positive children, 44.4% (12/27) had any allergic symptoms which showed a positive significant association (AOR = 2.787, 95% CI 1.253–6.197, P = 0.012) (Table 5).

**Table 5:** Potential risk factors for allergy among primary school children in Sululta, Ethiopia, 2017 (n = 526)

Variables	Total	Any allergy		COR	95% CI	P-value	AOR	95% CI	P-value
		Yes (%)	No (%)						
Sex									
Male	220	55 (25.0)	165 (75.0)	1.103	0.736–1.653	0.634			
Female	306	71 (23.2)	235(76.8)	1					
Age category									
5–9	158	33 (20.9)	125 (79.1)	1					
10–14	368	93 (25.3)	275 (74.7)	1.281	0.817–2.009	0.281			
Residence									
Urban	400	97 (24.3)	303 (75.7)	1.071	0.667–1.720	0.777			
Rural	126	29 (23)	97 (77)	1					
Animal									
Yes	346	85 (24.6)	261 (75.4)	1.104	0.721–1.690	0.648			
No	180	41 (22.8)	139 (77.2)	1					
Charcoal									
Yes	521	125 (24.0)	396 (76.0)	1.263	0.140–11.401	0.835			
No	5	1 (20.0)	4 (60.0)	1					
Electric									
Yes	174	48 (27.6)	126 (72.4)	1.338	0.882–2.030	0.171			
No	352	78 (22.2)	274 (77.8)	1					
Deworming									
Yes	427	94 (22.0)	333 (80.0)	0.596	0.368–0.957	0.032	0.591	0.366–0.955	0.034
No	99	32 (32.3)	67 (67.8)	1					
Any allergen									
Yes	27	12 (44.4)	15 (55.6)	2.702	1.229–5.937	0.013	2.787	1.253–6.197	0.012
No	499	114 (22.8)	385 (77.2)	1			1		
Parental allergy									
Yes	58	25 (56.9)	33 (43.1)	2.753	1.565–4.841	0.000	2.647	1.494–4.691	0.001
No	468	101 (21.6)	367 (78.4)	1					

Among children who did not take anthelminthic treatment within the last 6 months, 32.3% (32/99) reported having any allergic symptoms and in the deworming group, 22% (94/427) reported having any allergic symptoms. Recent anthelmintic treatment was inversely associated with self-reported allergic symptoms (AOR = 0.591, 95% CI 0.366–0.955, P = 0.034). Among the children with allergic symptoms, 56.9% (25/58) were from families who had a history of allergic symptoms, and this was positively related (AOR = 2.647, 95% CI 1.494–4.691, P = 0.001) (Table 5).

### Infection intensity of intestinal helminths

The intensity of helminthic infection was ranged between 24 and 1800 eggs per gram (Mean = 332) for *Ascaris lumbricoides*, 24 and 624 epg (Mean = 168) for hookworm, 24 and 144 (Mean = 69.78) for *Trichuris trichuria,* 24 and 720 (Mean = 106.0) for *Hymenolepis nana*, 48 and 48 (Mean = 48) for *Enterobius vermicularis*, 48 and 4800 (Mean = 645.82) for *Taenia spp*, and 48 (Mean = 48.0) for *Strongloid stercoralis*.

From 18 *Ascaris lumbricoides* positive children the egg count was performed for 15 children, from 33 *Hymenolepis nana* positive children the egg count was performed for 24 children and from 11*Trichuris trichuria* positive children the egg count was performed for 9 children. The egg count was not performed on the remaining children in each group because they were positive in the direct microscopy or in formol-ether concentration method but not in kato-katz method. The intensity of helminthic infection was based on WHO grading system [14]. All helminths eggs counted using kato-katz were light infection, and there was no significant association between infection intensity of *Ascaris lumbricoids*, Hookworm, *Hymnelopis nana* and *Tricuris tricuria* with allergen sensitization (P = 0.164, P = 0.554, P = 0.604) as shown in (Table 6).

**Table 6:** Association between helminthic infection intensity and Atopy in selected primary Schools in Sululta, Ethiopia, 2017(n = 526)

Helminths	Intensity (epg)	Atopy		COR	95% CI	P-value
		Yes (%)	No (%)			
*Ascaris*.*lumbricoids*	Light(1–4999)	2 (13.3)	13 (86.7)	2.991	0.640–13.979	0.164
	Negative	25 (4.9)	486 (95.1)	1		
	Mild (5000–49,999)	0	0			
	Heavy(> 50,000)	0	0			
*Hook worm*	Light(1–1999)	1 (9.1)	10 (90.9)	1.881	0.232–15.254	0.554
	Negative	26 (5.0)	489 (95.0)	1		
	Mild(2000–3999)	0				
	Heavy (> 4000)		0			
*Tricuris tricuria*	Light (1–999)	0	9 (100)			
	Negative	27 (5.2)	490 (94.8)			
	Mild (1000–9999)	0	0			
	Heavy (> 10,000)	0	0			
*Hymenolepis nana*	Light (1–1999)	0	24 (100%)	0.987	0.564–11.326	0.604
	Negative	27 (5.0)	502 (95.0)	1		
	Mild (2000–3999)	0				
	Heavy (> 4000)	0				

## Discussion

This study provides no evidence of significant association between overall helminthic infection and self-reported allergic symptoms. However, individuals infected with *Ascaris lumbricoides* had a significant positive association with atopy. Allergic symptoms were found to be inversely associated with recent anthelmintic treatment. The prevalence of atopy was found to be 5.1% and significantly related to any allergy symptoms. We also found a significant association between parental histories of allergic symptoms with childhood allergy disorders.

The prevalence of allergic symptoms in this study was relatively low compared with the report by Wordemann et al. in Cuban children (i.e. 21% for wheeze, 14% for rhinitis, 8% for eczema, and 20% for atopy) [7]. In contrast, the prevalence of all allergic symptoms, except atopy, in our study were higher than a study conducted by Cooper et al. in Ecuador which reported prevalence of 2.1%, 4.1%, 3.7%, and 18.2% for wheeze, rhinitis, eczema, and atopy [8]. These differences might be due to the differences in sample size and geographical location between the studies. The prevalence of atopy in our study was similar to the findings in a study in Jimma, Ethiopia, in which atopy prevalence was found to be 4.4% [22]. However, it was lower than the atopy prevalence reported in Mekele, Ethiopia, at 9.6% [23]. This difference may also be explained by differences in geographic location and sample size.

The finding of no significant association (P = 0.317) between helminthic infection and allergic symptoms is consistent with some [7, 27, 28] and contradictory to other previous studies. One possible explanation for the lack of association between helminthic infection and allergic disease in this study could be due to the recent implementation of a deworming program in the study site. This likely contributed to the low prevalence and low intensity of helminthic infection in our sample, which then could have affected the relationship between STH infection and allergic symptoms. More specifically, our finding was not in line with the studies conducted in Ethiopian immigrants in Israel and in Gabonese school children which both revealed significant inverse associations of helminthic infection and allergy [9, 12]. This inconsistency could be due to geographical location and low intensity of infection in our sample. In contrast, two cross-sectional studies in a Ugandan fishing community and in rural Bangladeshi children revealed that certain helminthic infections were positively associated with allergy-related outcomes [24, 25].

The finding that individual *Ascaris lumbricoides* infection had a significant positive association with atopy (P = 0.031) was consistent with previous findings, such as those conducted by Palmer et al. and Hawlader et al. [25, 26]. *Ascaris lumbricoides* infection has been linked to cross-reactivity towards environmental allergens, and therefore can increase skin prick test reactivity [27]. It is suggested that helminths do so by increasing the proliferation of Th2 cells which then causes an increased sensitization to allergens. However, in contrast to our results, other studies showed high parasite load and chronic infection might suppress atopy, while light and current infection could have the opposite effect [28]. Similarly, a randomized controlled trial conducted in Vietnam, showed that low parasite load increases allergen skin sensitization [29]. Several other studies also showed *Ascaris lumbricoides* infection showed inverse association with atopy [7, 11]. Possible reasons for this relationship could be due to the shared antigens between intestinal helminths and environmental allergens having a potential role in modulating allergic immune responses [30].

In our study, it was found that allergic symptoms were inversely associated with recent anthelminthic treatment (P = 0.034). This was in disagreement with previous studies in Cuban and Ecuador children which revealed deworming was not a risk factor for the development of allergic sensitization or for the atopic disease [31, 32]. There is no clear explanation for this association. However, the action of anthelminthic on allergic disease and host immune response after short-term deworming may contribute to this association. Further longitudinal studies are needed to understand the mechanism and observe the long-term effects of deworming on allergic sensitization and atopic disease.

Environmental allergens, particularly house dust mites and cockroaches have been associated with the development of childhood allergic disease [33]. Recent evidence indicated that 40–50% of schoolchildren have sensitization to one or more allergens [34]. In our study, we found that positive skin tests to cockroach antigens and house dust mite antigens to be 2.7% and 0.7% respectively. This report was lower than the previous study in Ethiopia (House dust mite, 10.8% vs. 8.2% Cockroach) [35]. Similarly, our finding was also lower than reports from Indonesia among primary school children, house dust mite, 7.03% and cockroach, 9.38% [36]. The possible explanation for this variation could be due to the large sample size in their study. We demonstrated that atopy had a positive significant association with any allergy symptoms (P = 0.012). This result was consistent with studies conducted in Uganda and Ecuador which revealed that atopy was associated with an increased risk of allergic symptoms [24, 37]. It was incongruent to a study conducted in Ethiopia that reported weak association between allergen sensitization and wheeze by Davey et al. [35].

Family history of allergy is considered as a risk factor for the development of childhood allergy, this is due to the fact that genetic in heritage has a great role in allergic disease [38, 39]. Previous research has shown that children born from atopic families showed an increased risk for the development of any type of allergies [22, 40]. We explored that parental history of allergic symptoms showed a statistically significant positive association with childhood allergy (P = 0.001), this was in agreement with a study conducted in Ethiopia by Amberbir et al. [41]*.*

The findings of this study however must be interpreted with caution, first the current study is cross-sectional design, which makes it difficult to attribute causality on the observed association since we didn’t have data on allergic disorder prior to parasite treatments. Research employing a longitudinal design is required in the future. Further limitation of this study was that only two allergen panels, German cockroach and dustmite, previously found to be common in an Ethiopia population were done [42]. It is possible our reported prevalence of atopy underestimated the true parameter.

## Conclusion

Our study provides no evidence of significant association between overall helminthic infection and self-reported allergic symptoms, which partly explained by lower intensity parasite infection due to the ongoing anthelmintic treatment. Effort to elucidate the relation between helminths and allergic disorder is potentially very important because a protective role not only indicates that helminths eradication from tropical populations will lead to epidemic increases in allergic disease but also raises the possibility that helminths products or even infection itself could be applied to therapeutic benefit.

## Data Availability

The datasets used and/or analyzed during the current study are available from the corresponding author on reasonable request.
